# Effects of dietary cold-pressed turnip rapeseed oil and butter on serum lipids, oxidized LDL and arterial elasticity in men with metabolic syndrome

**DOI:** 10.1186/1476-511X-9-137

**Published:** 2010-12-01

**Authors:** Ari Palomäki, Hanna Pohjantähti-Maaroos, Marja Wallenius, Päivi Kankkunen, Heikki Aro, Sari Husgafvel, Juha-Matti Pihlava, Kalevi Oksanen

**Affiliations:** 1 Kanta-Häme Central Hospital, Ahvenistontie 20, FI-13530, Hämeenlinna, Finland; 2 Linnan Klinikka, Raatihuoneenkatu 10, FI-13100, Hämeenlinna, Finland; 3 Heart Center, Kuopio University Hospital, P.O. Box 1777, FI-70211, Kuopio, Finland; 4 Mehiläinen, Sibeliuksenkatu 3, FI-13100, Hämeenlinna, Finland; 5 Finnish Institute of Occupational Health, Topeliuksenkatu 41 aA, FI-00250 Helsinki, Finland; 6 MTT Agrifood Research Finland, FI-31600, Jokioinen, Finland; 7 Finnish Funding Agency for Technology and Innovation, P.O. Box 69, FI-00101, Helsinki, Finland

## Abstract

**Background:**

Rapeseed oil is the principal dietary source of monounsaturated and n-3 polyunsaturated fatty acids in the Northern Europe. However, the effect of rapeseed oil on the markers of subclinical atherosclerosis is not known. The purpose of this study was to compare the effects of dietary intake of cold-pressed turnip rapeseed oil (CPTRO) and butter on serum lipids, oxidized LDL and arterial elasticity in men with metabolic syndrome.

**Methods:**

Thirty-seven men with metabolic syndrome completed an open and balanced crossover study. Treatment periods lasted for 6 to 8 weeks and they were separated from each other with an eight-week washout period. Subjects maintained their normal dietary habits and physical activity without major variations. The daily fat adjunct consisted either of 37.5 grams of butter or 35 mL of Virgino^R ^CPTRO. Participants were asked to spread butter on bread on the butter period and to drink CPTRO on the oil period. The fat adjunct was used as such without heating or frying.

**Results:**

Compared to butter, administration of CPTRO was followed by a reduction of total cholesterol by 8% (p < 0.001) and LDL cholesterol by 11% (p < 0.001). The level of oxidized LDL was 16% lower after oil period (p = 0.024). Minimal differences in arterial elasticity were not statistically significant.

**Conclusion:**

Cold-pressed turnip rapeseed oil had favourable effects on circulating LDL cholesterol and oxidized LDL, which may be important in the management of patients at high cardiovascular risk.

**Trial registration:**

ClinicalTrial.gov NCT01119690

## Background

Metabolic syndrome (MetS) is an international health problem. Risk factors included in the MetS are impaired glucose tolerance, elevated triglyceride levels, decreased HDL cholesterol concentration, elevated blood pressure and central obesity [1]. The risk of developing coronary heart disease is six to eight times greater and mortality in coronary heart disease two to three times greater among patients with MetS than among their healthy controls [2-5]. Furthermore, patients with MetS are at high risk to develop type 2 diabetes and myocardial infarction [4,5].

Atherosclerosis begins with an accumulation of lipoproteins, particularly low-density lipoprotein (LDL) into the intimae of arteries. In the arterial wall, LDL particles undergo oxidative modification which is suggested to play an important role in the atherosclerotic process [6]. Circulating oxidized LDL (oxLDL) seems to express the level of oxidative stress and associate with the risk factors of MetS [7]. Oxidized LDL has also been found to correlate with the extent of coronary heart disease and to be an independent predictor of an atherosclerotic plaque occurrence [8-10].

Oxidative modification of LDL damages the endothelium of the arterial wall [6]. Altered endothelial structure impairs the elasticity of the arteries already at an early stage of atherosclerosis [11]. Impaired elasticity of arteries may also result from factors affecting the elastic properties of the arterial wall, not only the endothelium. For example aging and hypertension may contribute to an overproduction of collagen and breaks in elastin fibers. Aortic stiffness has been reported to predict future coronary events and cardiovascular death in previous studies [12]. Thus, increased level of oxLDL and arterial stiffness might be used as early indicators of cardiovascular disease.

Despite of its' high total fat content, Mediterranean type diet rich in monounsaturated fatty acids (MUFA), decreases cardiovascular morbidity and mortality [13,14]. MUFA diminishes susceptibility of LDL to oxidation, which may contribute to this benefit [15-17]. The protective effect of n-3 polyunsaturated fatty acids (PUFA) from marine sources has been studied widely. However, the role of the plant derived n-3 PUFA, a principal dietary n-3 PUFA in the western diet, is less clear [18]. Rapeseed oil consists of high amounts of MUFA, a composition similar to that of olive oil. Rapeseed oil is also the main dietary source of plant-derived n-3 PUFA in the Northern Europe. However, the effect of rapeseed oil on the markers of subclinical atherosclerosis is not known.

Subjects with metabolic syndrome seem to have increased oxLDL levels and impaired arterial elasticity [19]. Manipulation of the fat quality in diet, to achieve alterations in plasma lipoproteins and arterial function, is an intriguing strategy in the prevention of atherosclerotic cardiovascular diseases. The aim of our study was to assess whether a dietary intake of cold-pressed turnip rapeseed oil (CPTRO) has beneficial effects on serum lipids, oxidized LDL and arterial elasticity compared to an intake of butter among men with metabolic syndrome.

## Methods

### Subjects

The present study is a part of Hämeenlinna Metabolic Syndrome research program HMS. It is a regional entity investigating atherosclerotic risk factors in men with MetS, who were referred from private and public consultations in primary and secondary health care. The inclusion criteria were male sex, age 30 to 65 years and a fulfilment of the criteria of MetS according to the National Cholesterol Education Program (NCEP) Adult Treatment Panel [1]. NCEP defines MetS as the presence of at least three of the following five criteria: 1) waist circumference > 102 cm, 2) serum triglycerides ≥ 1.7 mmol/L, 3) HDL cholesterol < 1.03 mmol/L, 4) blood pressure ≥ 130/85 mmHg and 5) fasting plasma glucose ≥ 6.1 mmol/L or diabetes.

43 study subjects, already recruited into HMS, participated in this dietary intervention study. Subjects were individually interviewed on their medical history, dietary habits, smoking and alcohol consumption. Physical examination consisted of height, weight, waist circumference and blood pressure measurements. Body mass index (BMI) was calculated as weight (kg)/height² (m²). Blood pressure was measured four times after at least ten minutes of rest in a semi-sitting position. The individual blood pressure was taken as a mean of these values. The weight and blood pressure measurements were repeated at the end of both study periods. Research personnel performing the laboratory and arterial elasticity measurements were blinded about the diet periods.

The Ethics Committee of the Kanta-Häme Hospital District approved the study protocol and the study followed the ethical principles outlined in the Declaration of Helsinki. Each study subject gave a written informed consent.

### Design and Diets

**Figure 1:**
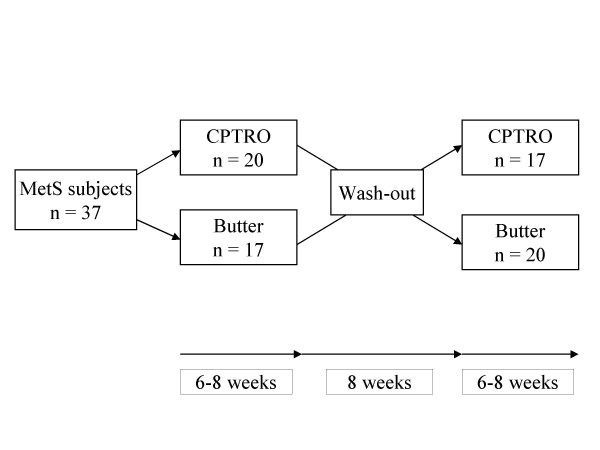
**Design of the open, randomized cross-over study**. Six of 43 subjects withdrew during the study because they were unable to comply with the dietary regimens.

Subjects' usual diet was supplemented with CPTRO and butter in an open, balanced and randomized crossover design. The study consisted of two study periods (oil and butter) lasting from 6 to 8 weeks, and they were separated from each other by an 8-week washout period (Figure 1). To avoid the effect of seasonal variation on the results, the first study period occurred from October to December and the second period from January to March.

At the beginning of each fat supplementation period the participants were given both verbal and written detailed instructions to be followed. Butter with a maximal water content of 16% and cold-pressed turnip rapeseed oil (0.92 g/ml) were provided free of charge. The daily fat adjunct consisted either of 37.5 grams of butter (Valio Ltd, Finland) on the saturated fatty acid (SFA) period and 35 mL of Virgino^R ^CPTRO (Kankaisten Öljykasvit Ltd) on the oil period, which comprised approximately 11-12% of daily energy intake. We recommended participants to use the fat adjunct as such, without heating or frying. To keep the diets as stable as possible, participants were asked to spread the butter supplement on bread during the butter period and to drink the oil supplement as well as to eat their bread without spread during the oil period. Besides these instructions subjects were asked to maintain their normal dietary habits and physical activity without major variations. Heating or frying of other fat included in the subjects' every-day diet was not forbidden. To ensure that subjects' everyday diet and physical activity remained similar during both periods, they were asked to fill in a structured questionnaire on their diet as well as on the amount, intensity and type of physical exercise during the study.

### Analysis of Fatty Acids and Phenolic Compounds

The fatty acid compositions of cold-pressed turnip rapeseed oil and butter were analysed at MTT Agrifood Research Finland (Jokioinen, Finland). Fatty acids were analysed as their methyl esters. The analysis was carried out using a Hewlett Packard 5890 capillary gas chromatograph equipped with a DB-23 column coated with a cyanopropyl-stationary phase (60 m * 0.25 mm id, film thickness 0.25 μm) and a mass selective detector. A mixture of standard fatty acids (C4:0 - C24:1) purchased from Nu-Check-Prep Inc. (Minnesota, USA) was used as the standard to identify the peaks.

To analyze the amount of phenolic compounds, sample of CPTRO was dissolved into hexane and extracted with 80% methanol according to a modified method of Vuorela et al [20]. Phenolic compounds were identified and quantified by high performance liquid chromatography with diode array detection using analytical conditions similar to those used for the determination of alkylresorcinols [21]. Vinylsyringol (2,6-dimethoxy-4-vinylpheol) was quantified as syringic acid.

The fatty acid compositions of the dietary fat supplements i.e. Virgino^R ^cold-pressed turnip rapeseed oil and butter are shown in Table 1. Butter consisted mainly of saturated fatty acids (61.7%). Oleic acid was the main unsaturated fatty acid found in butter. CPTRO consisted mainly of unsaturated fatty acids (95.6%), over half of which were monounsaturated ones. The major fatty acids in oil were oleic acid, linoleic acid and alfa-linolenic acid. CPTRO contained also a small amount of phenolic compounds. Total amount of sinapine and sinapic acid was 0.94 μmol/100 g and of vinylsyringol 0.89 μmol/100 g. Also some smaller peaks of unknown phenolic compounds were seen in the high performance liquid chromatograms.

**Table 1:** Distribution of fatty acids in cold-pressed VirginoR turnip rapeseed oil (CPTRO) and butter used in the study

Fatty acids	Distribution %	
	CPTRO	Butter
10:0 capric acid	0	1.6
12:0 lauric acid	0	2.7
14:0 myristic acid	0	11.0
14:1 (ω-5) myristoleic acid	0	0.9
15:0 pentadecanoic acid	0	0.9
16:0 palmitic acid	2.9	30.0
16:1 (ω-7) palmitoleicacid	0.1	1.5
18:0 stearic acid	1.3	14.6
18:1 (ω-7) vaccenic acid	2.9	0.9
18:1 (ω-9) oleic acid	56.7	30.4
18:2 (ω-6) linoleic acid	21.9	2.0
18:3 (ω-3) alpha-linolenic acid	13.1	0.4
20:1 (ω-9) eicosenoic acid	0.9	0
Others (8:0, 17:0, 18:2, 20:0)	0.3	1.4
Unidentified fatty acids	0	1.8
Saturated fatty acids	4.5	61.7
Monounsaturated fatty acids	60.6	33.7
Polyunsaturated fatty acids	35.0	2.9

The following fatty acids analyzed did not exist in CPTRO nor in butter: 16:0(i), 16:1, 17:1, 18:3 (ω-6), 18:4 (ω-3), 18:4 (ω-3), 19:1, 20:2 (ω-6), 20:3, 20:3 (ω-3), 20:4 (ω-6), 20:5 (ω-3), 22:0, 22:1 (ω-9), 22:2, 22:3, 22:4 (ω-6), 22:5 (ω-3), 22:6 (ω-3), 24:0, and 24:1 (ω-9).

### Blood Sampling and Biochemical Analysis

At the end of both dietary periods, blood samples were collected into 10 mL EDTA tubes, 5 mL lithium-heparin gel tubes and 2 mL natrium-fluoride tubes after a 12-hour overnight fast and at least ten minutes of rest. Levels of HDL, LDL and total cholesterol and triglycerides as well as glucose and HbA1C were analyzed by Cobas Integra procedure (Roche). HbA1C was assessed in % and conversed to mmol/mol [22]. The laboratory practices strict internal quality control with daily and monthly control samples performed by a national external quality assurance program (Labquality Oy).

### Determination of Oxidized LDL

Plasma levels of oxidized LDL were determined as duplicates after both diet intervention periods. Determinations were made according to a validated monoclonal antibody 4E6-based capture ELISA (Mercodia AB, Uppsala, Sweden) [7,9]. The monoclonal antibody mAb-4E6 is the same as in the assays previously described by Holvoet et al [8,10]. Samples were diluted with sample buffer in two steps to gain a final dilution 1/6561. 25 μl of each calibrator, control and diluted sample were pipetted into wells containing mouse monoclonal anti-oxidized LDL. 100 μl assay buffer was added into each well. Samples were washed 6 times with automatic washer before 100 μl peroxidase conjugated mouse monoclonal anti-apoB was added. After a 60-minute incubation at room temperature, samples were washed again and the bound conjugate was detected by reaction with 200 μl 3,3', 5,5'-tetramethylbenzidine. Reaction was stopped by 50 μl 0.5 M H_2_SO_4 _and the colorimetric endpoint was read spectrophotometrically at 450 nm. Same experienced researcher at Kanta-Häme Central Hospital performed all determinations of oxidized LDL.

### Determination of Arterial Elasticity

An experienced nurse measured the elasticity of large and small arteries after at least 10 minutes of rest in a semi-sitting position. The recording was carried out in a temperature-controlled room after an overnight fast. Subjects were asked to refrain from eating, smoking, drinking caffeinated drinks and taking medication for 12 hours and drinking alcohol for two days prior to measurement. Radial artery pulse wave was recorded non-invasively with an arterial tonometry (HDI/PulseWave™CR-2000) that uses a modified Windkessel pulse-contour method [11]. The capacitive elasticity of large arteries (C1) and the reflective elasticity of small arteries (C2) were automatically assessed as a mean of five most similar pulse waves appearing during thirty seconds of measurement. Four measurements were performed to gain mean large and small arterial elasticity for every subject. Studies were performed at the end of both diet periods.

### Statistical Analysis

Statistical analysis was carried out with the SPSS software (version 17.0, SPSS 2009). Basic results are reported as mean ± SD. Paired samples' T-test was used to compare the outcome measurements after diets in case of normality. Wilcoxon's test was used in case of non-normality. These results are reported as mean ± SEM. Statistical significance was accepted at p value < 0.05.

## Results

Six men withdrew because they were unable to comply with the dietary regimens. Results include thirty-seven subjects who completed both study periods. Baseline characteristics of these 37 men are shown in Table 2.

**Table 2:** Basic clinical and laboratory characteristics of 37 men with metabolic syndrome accomplishing all clinical phases of the study

	Mean (SD)	Range
Height, cm	177.1 (5.7)	163.0 - 191.0
Weight, kg	97.4 (16.3)	68.0 - 142.0
Waist circumference, cm	111.1 (13.1)	92.0 - 150.0
BMI, kg/m^2^	31.0 (5.0)	23.9 - 46.4
SBP, mmHg	145.5 (12.3)	122.0 - 172.0
DBP, mmHg	90.8 (5.6)	80.0 - 105.0
Fasting glucose, mmol/L	6.62 (1.19)	3.3 - 9.5
HbA1C		
(%)	6.42 (0.66)	5.3 - 8.7
(mmol/mol)	46.7 (7.2)	34.4 - 71.6
Total cholesterol, mmol/L	5.15 (1.49)	2.6 - 8.4
HDL-C, mmol/L	1.15 (0.27)	0.5 - 1.7
LDL-C, mmol/L	3.33 (1.25)	1.1 - 6.0
Triglycerides, mmol/L	2.19 (1.13)	0.9 - 6.3
ALAT, U/L	53.0 (27.4)	25.0 - 163.0
GT, U/L	72.4 (62.9)	15.0 - 275.0
Afos, U/L	67.6 (19.7)	30.0 - 115.0
Fibrinogen, g/L	3.49 (0.96)	2.3 - 5.5

There were six current smokers, twenty-one ex-smokers and nine non-smokers. Twenty-one men had earlier been diagnosed with hypertension, nineteen with type 2 diabetes and six with coronary heart disease. Pharmacological therapy consisted of aspirin or other antiplatelet agent in fourteen, beta-blockers in sixteen, and ACE inhibitors or angiotensin_1 _receptor inhibitors in fourteen subjects, but only four were on glucose-lowering drugs. Fifteen men were on statin medication, two of these on combination therapy of statin and fibrate.

**Figure 2:**
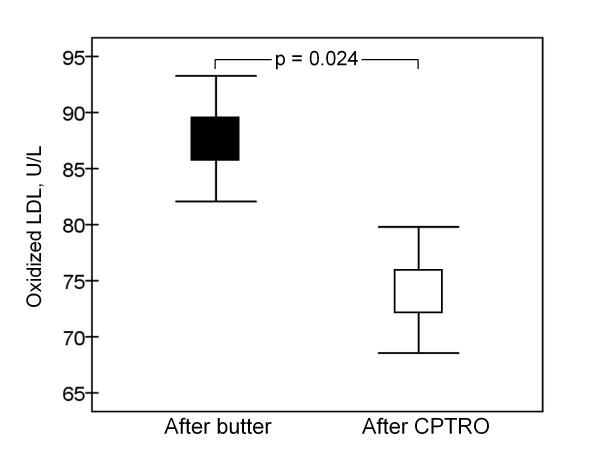
**Plasma concentration of oxidized LDL at the end of fat supplementation periods**. OxLDL was 87.7 ± 5.6 U/L after the butter and 74.2 ± 5.6 U/L after the CPTRO (cold-pressed turnip rapeseed oil) period (p = 0.024).

**Figure 3:**
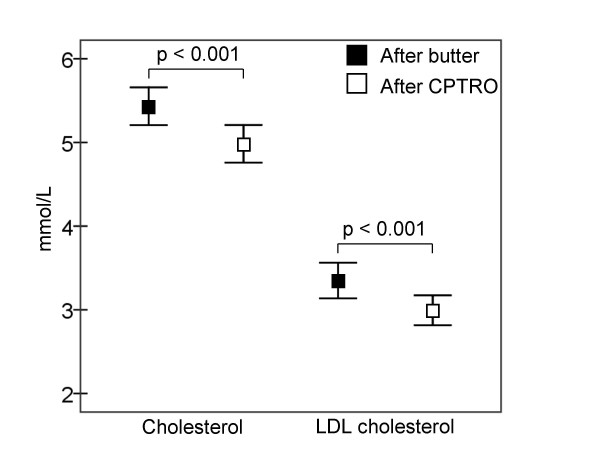
**Concentrations of total and LDL cholesterol**. Total cholesterol was 5.43 ± 0.23 mmol/L at the end of the butter and 4.98 ± 0.23 mmol/L at the end of the CPTRO (cold-pressed turnip rapeseed oil) period (p < 0.001). LDL cholesterol was 3.35 ± 0.21 mmol/L and 3.00 ± 0.18 mmol/L, respectively (p < 0.001).

Physical activity, medication and every-day diet of participants, evaluated by a structured questionnaire, remained stable during the study. As presented in Figure 2, the level of oxidized LDL was significantly lower (16%) after the cold-pressed turnip rapeseed oil period compared to the butter period (p = 0.024). Total and LDL cholesterol were also significantly lower after the CPTRO period compared to the levels after the butter period, 8% (p < 0.001) and 11% (p < 0.001), respectively (Figure 3). No significant differences in HDL cholesterol, triglycerides, glucose or HbA1C were found between the periods. The concentration of HDL cholesterol was 1.15 ± 0.04 mmol/L after both diet periods. Triglycerides were 2.72 ± 0.30 mmol/L after the butter and 2.56 ± 0.38 mmol/L after the CPTRO periods (NS).

There were no significant differences in the mean large or small arterial elasticity between the diet periods (Table 3). Systolic blood pressure was 138.5 ± 2.2 mmHg after the butter and 138.5 ± 2.6 mmHg after the CPTRO period. Diastolic blood pressures were 81.8 ± 1.4 mmHg and 81.3 ± 1.6 mmHg, respectively. Weight remained stable through the study, 97.1 ± 2.8 after the butter and 97.1 ± 2.7 after the oil period. Total blood count showed no significant differences between rapeseed oil and butter consumption except in the mean corpuscular haemoglobin (31.1 ± 0.2 pg vs. 30.8 ± 0.2 pg, p = 0.01) and in the platelet count (221.6 ± 9.0 E9/L vs. 235.4 ± 9.5 E9/L, p = 0.003).

**Table 3:** Large (C1) and small (C2) arterial elasticity at the end of butter and oil periods.

	Butter period	Oil period	p value
C1, mL/mmHg × 10	15.5 ± 0.46	15.9 ± 0.71	NS
C2, mL/mmHg × 100	6.26 ± 0.48	6.34 ± 0.44	NS

Data are presented as mean ± SEM

## Discussion

In this study among men with MetS dietary cold-pressed turnip rapeseed oil was associated with favourable changes in lipid parameters compared to the use of butter. The lower level of total cholesterol after CPTRO consumption was due to the lower level of LDL cholesterol. However, the concentration of circulating oxLDL was proportionally even lower than that of LDL cholesterol after the CPTRO period.

To our knowledge, this is the first report on the beneficial effect of rapeseed oil on circulating oxLDL levels. The significant reductions in total and LDL cholesterol levels are in accordance with earlier rapeseed oil studies [23,24]. In agreement with most of the previous studies [23,25,26] the use of rapeseed oil did not have significant effect on HDL cholesterol levels, although a slight increase in HDL cholesterol by rapeseed oil has also been reported [24].

LDL particles enriched with MUFA seem to be resistant against oxidation [15-17]. Since MUFA are the main fatty acids in rapeseed oil, this protective effect may contribute to the decreased level of oxLDL after CPTRO enriched diet in the present study. Previously, a diet with high content of MUFA has been shown to decrease oxLDL levels among healthy non-obese men as well as among subjects at high cardiovascular risk [27,28]. Although effects of turnip rapeseed oil among metabolic syndrome patients have not been studied earlier, high MUFA-rich diet has been reported to improve oxidative stress parameters in patients with MetS [29]. Also in a study by Perona et al [30], MUFA-rich virgin olive oil protected LDL against oxidation among type 2 diabetics.

The origin of circulating oxLDL is unknown. Oxidative modification of LDL can generate minimally or fully oxidized LDL [31]. It is generally thought that oxidation of LDL occurs mainly in the arterial wall and only minimally oxidized LDL exists in the circulation [6,31]. However, increased circulating oxLDL, determined by a method using the same monoclonal antibody as in the present study, is known to associate both with subclinical and clinical atherosclerosis as well as to predict future cardiovascular events [8,9,32]. Thus, dietary manipulations that protect LDL against oxidation may delay development of atherosclerotic lesions in patients with MetS.

Increased glucose levels, often present among subjects with MetS, may enhance the oxidation of lipoprotein(a) [33]. Previously, a low-carbohydrate (LC) diet had more favourable effects on MetS features compared to a low-fat diet [34]. A low-carbohydrate diet has also been reported to prevent the accumulation of oxLDL in the arterial wall [35]. Whether a LC-diet supplemented with CPTRO would cause even greater reductions in oxLDL and prevent future cardiovascular events among MetS subjects would be interesting to study.

Most dietary approaches to prevent hyperlipidaemia and atherosclerosis have focused on the alteration of fat composition and quantity in diet. In the present study, CPTRO had a cholesterol-lowering effect as a supplement to the habitual diet. Turnip rapeseed oil is the main Finnish source of unsaturated fatty acids, mainly monounsaturated ones. By improved cultivation, MUFA in rapeseed oil consist of an important amount of oleic acid. Mediterranean type diet, also rich in MUFA, has been shown to decrease total cardiovascular mortality [13]. Compared to olive oil, common in the Mediterranean region, Virgino^R ^rapeseed oil contains less MUFA and SFA but a considerable amount of n-3 PUFA, namely alfa-linolenic acid (see Table 1).

Low dose of n-3 PUFA supplement reduced early mortality after myocardial infarction in the GISSI-Prevenzione trial [36]. On the other hand LDL susceptibility to oxidation has been assumed to associate with an increased amount of PUFA in the small and dense LDL particles [37]. In addition, PUFA-rich diet increased susceptibility of LDL to oxidation when compared to MUFA-rich diet among subjects with impaired glucose intolerance [38]. However, moderate amounts of highly unsaturated n-3 fatty acids in a diet supplemented with rapeseed oil did not increase LDL oxidizability when provided in the context of a diet rich in MUFA [15].

In addition to MUFA, phenolic compounds of olive oil have been shown to reduce oxidative damage [39]. Rapeseed oil, supplemented in the present study, contained only slight amounts of phenolic compounds. Therefore, the antioxidative effect of rapeseed oil is presumably mediated mainly by the high contents of MUFA and n-3-PUFA.

In our study, arterial elasticity seemed to be minimally better after the CPTRO than after the butter period, but the difference was not statistically significant. One explanation might be that the intervention period was too short to affect the function of the arterial wall. However, McVeigh et al [40] have found improved arterial compliance already after a 6-week fish oil administration in patients with non-insulin-dependent diabetes. Arterial stiffness is not merely affected by endothelial dysfunction. For example, untreated long-lasting hypertension and aging may cause increase in the amount of collagen and decrease in the amount of elastin fibers in the arterial wall [12]. Alterations in the elastic properties of the arterial wall contribute to the impairment of elasticity.

Since endothelial dysfunction is a key mechanism in atherosclerosis, a measurement of mere endothelial function might be more sensitive in a short-term intervention study [11]. Previously, a walnut diet rich in antioxidants and alfa-linolenic acid for four weeks showed an improvement in endothelial function of hypercholesterolaemic subjects [41]. Even one meal supplemented with walnuts rich in n-3-PUFA, has been reported to improve flow-mediated dilation of arteries as a sign of improved endothelial function [42]. The same effect was however not seen after a meal supplemented with MUFA-rich olive oil [42]. Differences in fat type and study groups or methods used to evaluate arterial function might explain the seeming discrepancy of the results between the abovementioned studies and the present one. It would be interesting to study the effects of rapeseed oil among subjects with evidently decreased arterial elasticity.

The platelet count was lower after the CPTRO period in our study. It has been revealed earlier that fish oils decrease fibrinogen level and platelet count [43]. Vegetable n-3 PUFA, like alfa-linolenic acid, is an important constituent of the Virgino^R ^CPTRO. During fat metabolism it can be converted to longer chain fatty acids found also in fish oils. Hence, the significant decrease in platelet count after CPTRO consumption may be attributed to the n-3 PUFA.

Strength of this study, unlike of many other dietary studies, was the balanced crossover design used to diminish the variability between the subjects. We did not measure the fat and energy intake by dietary records, which was a limitation of the study. However, we ensured the stability of both habitual diet and physical activity during the study periods by analyzing the detailed questionnaire that subjects filled in at the end of the intervention periods. Previously a similar food frequency questionnaire has been reported to be a suitable tool for ranking energy and nutrient intake [44]. In addition, there was no difference in weight between the two dietary periods which is in agreement with previous studies on n-3-PUFA and MUFA rich diets [45,46]. Hence, the difference in energy balance did not explain our findings.

A healthy cohort would have been an interesting addition to the study. However, we have previously reported increased oxLDL levels as well as impaired large arterial elasticity among MetS subjects compared to their healthy, age-matched counterparts [19]. Therefore, in the present study we wanted to focus on MetS subjects because of their increased risk for atherosclerotic cardiovascular diseases.

Vegetable oils are often consumed in cooking and frying. However, repeated heating may destroy the beneficial compounds of the oils and generate unhealthy effects instead [47,48]. Therefore, study subjects were asked to drink CPTRO as such, without heating or frying. Heating or frying of fat included in the subjects' every-day diet was not forbidden. We cannot determine the optimal daily dose of CPTRO based on the results of this study, in which a constant dose of CPTRO and a comparable amount of butter were used. Previously whipped cream with a similar dose of SFA as used in the present study did not cause a significant increase in postprandial oxidative stress [49]. Thus, at least the dose of CPTRO, used in the present study, seems to be beneficial as a supplement to a diet. In practise, it may be used as such for example in salad and dip sauces. It is worth to notice that the study included only men with MetS. Hence, the results cannot necessarily be generalized to the whole population including women and men without MetS.

## Conclusion

A short-time modification of the diet with cold-pressed turnip rapeseed oil compared to butter decreased the concentration of circulating LDL cholesterol and oxidized LDL. In men with metabolic syndrome, modification of the diet with cold-pressed turnip rapeseed oil may be beneficial in treating hyperlipidaemia and diminishing oxidative stress and thereby delaying the progression of atherosclerosis.

## List of Abbreviations

BMI: body mass index; C1: large arterial elasticity; C2: small arterial elasticity; CPTRO: cold-pressed turnip rapeseed oil; HbA1C: glycosylated haemoglobin; HDL: high density lipoprotein; HMS: Hämeenlinna Metabolic Syndrome Research Program; LC: low-carbohydrate; LDL: low density lipoprotein; MetS: metabolic syndrome; MUFA: mono-unsaturated fatty acids; NCEP: National Cholesterol Education Program; oxLDL: oxidized low density lipoprotein; PUFA: poly-unsaturated fatty acids; SFA: saturated fatty acids

## Competing interests

The authors declare that they have no competing interests.

## Authors' contributions

AP designed the study with the help of KO. AP, HPM and MW participated in the acquisition of data, analysis and drafting of the manuscript. PK, HA, SH, and JMP made a substantial contribution in the acquisition of data and helped in drafting the manuscript, KO participated in the acquisition of data and gave the final approval of the version to be published. All authors read and approved the final manuscript.
